# Emerging approaches to target mitochondrial apoptosis in cancer cells

**DOI:** 10.12688/f1000research.18872.1

**Published:** 2019-10-24

**Authors:** Andrew Gilmore, Louise King

**Affiliations:** 1 Wellcome Centre for Cell-Matrix Research, Faculty of Biology, Medicine and Health, Manchester Academic Health Science Centre, University of Manchester, Manchester, UK

**Keywords:** Apoptosis, BH3-mimetics, cancer

## Abstract

Apoptosis is a highly conserved programme for removing damaged and unwanted cells. Apoptosis in most cells is coordinated on mitochondria by the Bcl-2 family of proteins. The balance between pro- and anti-apoptotic Bcl-2 family proteins sets a threshold for mitochondrial apoptosis, a balance that is altered during cancer progression. Consequently, avoidance of cell death is an established cancer hallmark. Although there is a general perception that tumour cells are more resistant to apoptosis than their normal counterparts, the realities of cell death regulation in cancer are more nuanced. In this review we discuss how a profound understanding of this control has led to new therapeutic approaches, including the new class of BH3-mimetics, which directly target apoptosis as a vulnerability in cancer. We discuss recent findings that highlight the current limitations in our understanding of apoptosis and how these novel therapeutics work.

## Introduction

An important issue in cancer therapy is how to target tumour cell populations whilst minimising impact on healthy surrounding cells. Dose-limiting toxicity is a major problem restricting the treatment to patients, particularly with the types of cytotoxic agents used for more advanced tumours. These types of drugs can be as harmful to many healthy tissues as they are to the tumour cells. Cytotoxic chemotherapy kills cancer cells by inducing apoptosis. Although resistance to apoptosis is an established cancer hallmark, how sensitivity differs between tumours and normal tissues is a more complex and nuanced issue than simply that cancer cells are more resistant to cell death. The increased understanding of apoptosis that has developed over the past decades is now providing new opportunities for targeting vulnerabilities in cancer.

The concept of selectively activating cell death in cancer cells has a long history ^1^. Programmed cell death can occur via a number of mechanisms, including apoptosis and necroptosis, regulated by distinct signalling pathways. A number of these cell death signalling pathways have been exploited with varying success as potential therapeutic targets ^2, 3^. The disappointing early clinical results with some approaches have been due, in part, to an incomplete understanding of the underlying biology. The successful development of venetoclax highlights how understanding the underlying mechanisms allows rational drug design targeted specifically at apoptotic regulators ^4^. In this review, we examine the successful exploitation of mitochondrial apoptosis to develop these novel therapeutic approaches and we discuss some of the potential future issues surrounding them.

## Bcl-2 proteins: setting the apoptotic landscape for cell death

**Figure 1.**
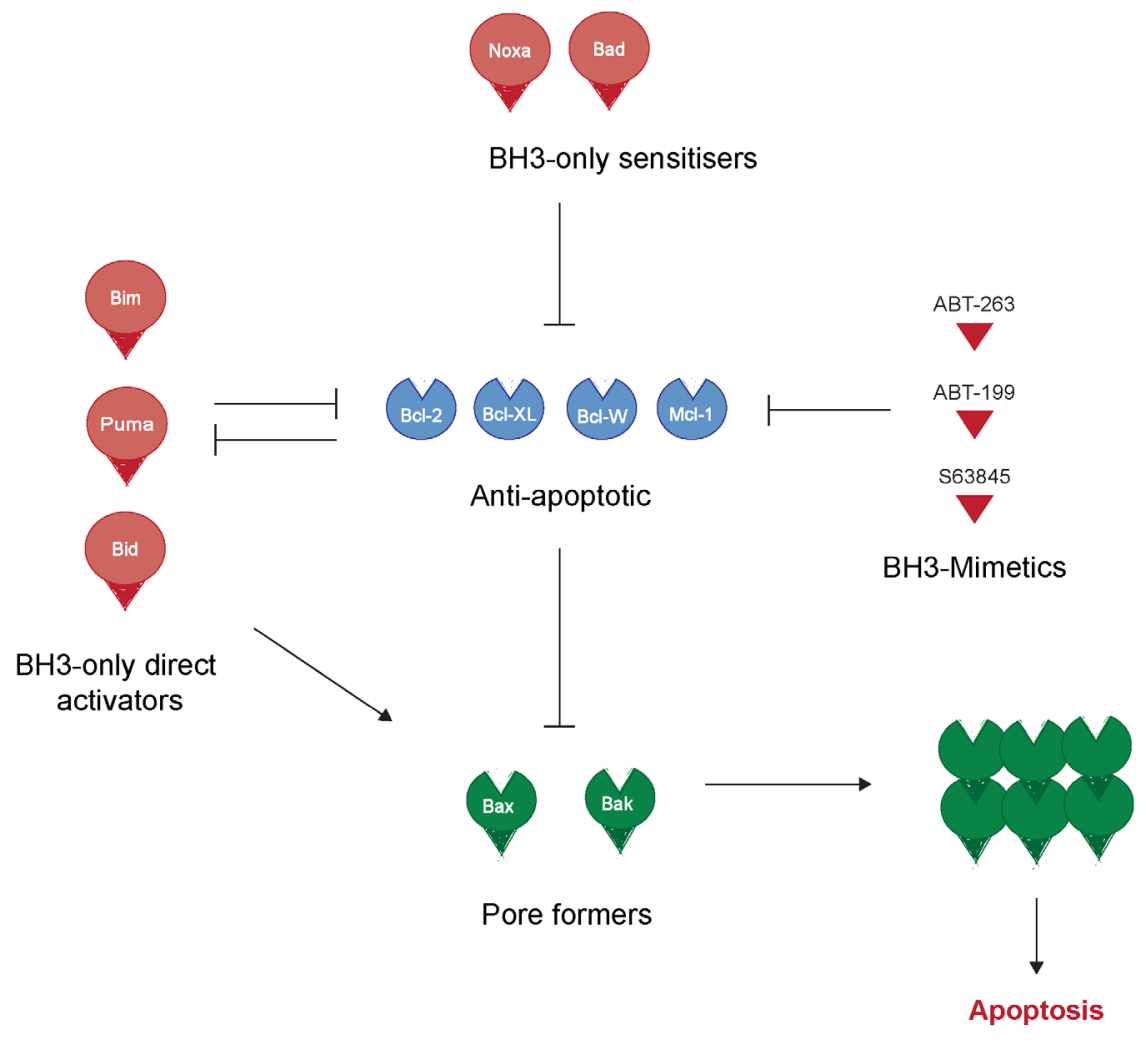
The canonical interactions between Bcl-2 family protein subgroups. The Bcl-2 family of proteins consists of three groups: anti-apoptotic proteins (for example, Bcl-2, Bcl-XL, Bcl-W and Mcl-1), pore-forming pro-apoptotic proteins (for example, Bax and Bak) and the BH3-only proteins. The BH3-only subgroup shows distinct binding preferences for both anti- and pro-apoptotic Bcl-2 proteins. Some BH3-only proteins, such as Noxa and Bad, bind only specific anti-apoptotic proteins. As such, they do not directly activate Bax and Bak and are termed “sensitizer” BH3-only proteins. Other BH3-only proteins, including Bim, Bid and PUMA, can bind both anti- and pro-apoptotic proteins. These either can activate pro-apoptotic Bax and Bak (and thus are termed “direct activators”) or can be inhibited by binding the anti-apoptotic proteins. The BH3-domain of the BH3-only proteins represents a canonical site of interaction with the other subgroups. BH3-mimetics such as ABT-263, ABT-199 and S63845 have been developed to mimic the interaction of specific BH3-only proteins with anti-apoptotic proteins.

*BCL-2* was initially identified at the site of the t(14;18) chromosomal translocation in patients with B-cell lymphoma ^5^. The positioning of *BCL-2* at the translocation junction near the immunoglobulin locus altered its transcriptional regulation but this did not drive proliferation like other oncogenes ^6, 7^. The realisation that Bcl-2 protein overexpression contributed to oncogenesis by inhibiting programmed cell death kick-started studies leading to the identification of a family of apoptosis regulators ^8^ and established evasion of apoptosis as a central hallmark of cancer ^9^. Now, nearly 20 members of the Bcl-2 protein family have been confirmed in vertebrates ^10^, and the multitude of interactions between these proteins is central to how both normal and cancer cells respond to cytotoxic damage ( Figure 1).

Members of the Bcl-2 family can be characterised by sharing at least one homologous region within their sequence, termed Bcl-2 homology or BH-domains ^10^. Proteins within the family can be grouped based on both the presence of these BH-domains and their function in apoptosis regulation. Bcl-2, along with Bcl-XL, Mcl-1, Bcl-W and A1, are anti-apoptotic and contain four distinct BH-domains (sometimes referred to as BH1–4 proteins). These anti-apoptotic proteins are responsible for binding pro-apoptotic Bcl-2 proteins to inhibit their function. The pro-apoptotic Bcl-2 proteins can be further categorised on the basis of function and sequence homology. Like the anti-apoptotic members, the effector proteins Bax, Bak and Bok also have multiple BH-domains. Bax and Bak are the best understood. Both promote apoptosis by effecting mitochondrial outer membrane permeabilisation (MOMP), releasing pro-apoptotic factors such as cytochrome *c* and SMAC/Diablo. Bax and Bak, along with the anti-apoptotic proteins, also have a C-terminal tail anchor region that targets these proteins to membranes, predominately (though not exclusively) to mitochondria. The role of Bok is less understood, and although it does share homology with Bax and Bak, Bok appears to be predominantly regulated through proteasomal degradation at the endoplasmic reticulum ^11^.

The final group of Bcl-2 proteins, termed BH3-only proteins because they share only the single region of homology with the other family members, are the most diverse ^12^. Proteins in this group include Bid, Bad, Bim, Noxa, PUMA, Bmf, Hrk and Bik. These can bind directly to both pro- and anti-apoptotic multidomain proteins via their BH3-domain, which comprises a short amphipathic α-helix. This binding can either inhibit the anti-apoptotic proteins or directly activate pro-apoptotic Bax and Bak. Differences in the sequence of BH3-domains mean that different BH3-only proteins have distinct binding specificities for different multidomain proteins. Bim and Bid are promiscuous, binding most pro- and anti-apoptotic proteins, whereas Bad binds only Bcl-2, Bcl-XL and Bcl-W, and Noxa binds just Mcl-1 and A1.

The final component contributing to the complexity of apoptosis comprises the plethora of signals controlling both transcriptional and post-translational regulation of the different Bcl-2 family members. The details of these regulatory mechanisms are reviewed in detail elsewhere ^12^; however, examples relevant to cancer include the transcriptional activation of PUMA and Noxa by the p53 tumour suppressor ^13, 14^, potentially linking these BH3-only proteins to chemotherapies inducing genotoxic stress. Other BH3-only proteins are post-translationally regulated; these include Bad, which undergoes phosphorylation on multiple sites in response to the types of growth factor signalling upregulated by many oncogenic mutations in cancer ^15^. The key concept is that a cell will be expressing a range of pro- and anti-apoptotic Bcl-2 proteins that together set a threshold above which further cytotoxic damage will result in MOMP.

**Figure 2.**
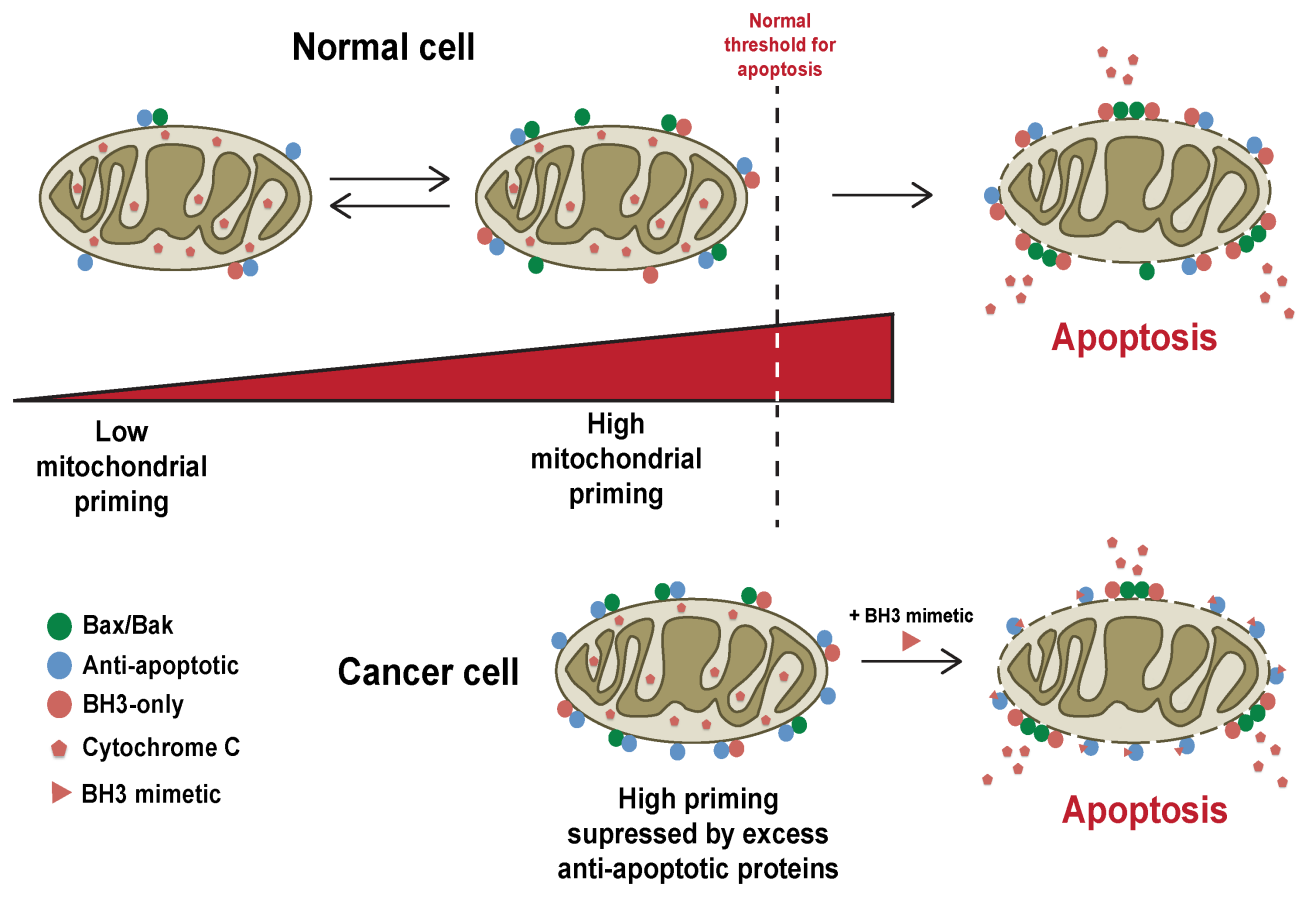
Apoptotic priming describes the proximity of a cell to the point of commitment to mitochondrial outer membrane permeabilisation (MOMP). Cells can exist in different states of priming, which essentially describes how close the cell lies to MOMP, which is dependent upon the functional Bcl-2 protein landscape in that cell. Normal cells can shift their state of priming following changes in cellular stress or exposure to pro-apoptotic stimuli, such as DNA damage or growth factor deprivation. These signals result in changes in the degree of priming, although this may not result in MOMP. Normal cells are often insensitive to BH3-mimetics. In contrast, cancer cells are often primed and closer to MOMP. Overexpression of anti-apoptotic Bcl-2 proteins in cancer cells can suppress MOMP by sequestering pro-apoptotic Bcl-2 family members. However, this results in them being extremely sensitive to BH3-mimetics, which can compete with the pro-apoptotic proteins, releasing them from their anti-apoptotic partners to drive MOMP ^37^.

This intricate balance of interactions between the pro- and anti-apoptotic Bcl-2 proteins allows them to act as a buffer against the constantly changing signals reaching the cell, keeping healthy cells alive whilst killing any cells that either become too damaged or are surplus to requirement. As cells acquire or become exposed to damage, the complex Bcl-2 landscape shifts through changes in the expression or function of pro-apoptotic members and downregulation or neutralisation of anti-apoptotic ones ( Figure 2). These changes make cells more primed to undergo MOMP. Conversely, alterations such as overexpression of anti-apoptotic proteins make cells less primed. Therefore, it is unsurprising that cancer cells show alteration in their Bcl-2 landscape.

## Overexpression of Bcl-2: a paradigm for cancer cells altering the apoptotic landscape

Since the original characterisation of the (14;18) translocation in lymphoma, Bcl-2 protein expression has been found to be elevated in a range of cancers, including chronic lymphocytic leukaemia (CLL) ^16^, small cell lung carcinoma (SCLC) ^17^ and neuroblastoma ^18^. However, translocations or mutations within anti-apoptotic Bcl-2 family genes in general are the exception for how these become dysregulated in cancer. More common are gene amplifications or increased protein expression, the latter occurring through increased transcription, translation or protein stability. Of the anti-apoptotic members of the family, *BCL2L1* (encoding Bcl-XL) and *MCL1* are the most frequent genes amplified in human cancers ^19^. The consequences of anti-apoptotic Bcl-2 protein overexpression will be to increase the threshold for the level of damage required to induce MOMP. This functional outcome is seen in the multitude of studies where the expression of these proteins has been artificially manipulated, be it in cells or mice.

Although overexpression of anti-apoptotic Bcl-2 proteins is seen in many cancers, by itself this is only weakly oncogenic, if at all. Indeed, the (14;18) translocation involving *BCL2* has also been seen in healthy lymphocytes ^20^. When overexpression of anti-apoptotic Bcl-2 proteins has been engineered into mouse models, it has not by itself been profoundly oncogenic, and any predisposition to tumours seen tends to have a long latency ^21^. Amplification of anti-apoptotic Bcl-2 protein expression, however, does combine potently with other oncogenes, such as Myc, and this is seen in human tumours as well as in animal models and cell lines ^19, 22– 25^. Mcl-1 can also undergo co-amplification with Myc in breast cancer ^26^.

Does anti-apoptotic Bcl-2 protein overexpression make cancer cells more resistant to apoptosis than healthy cells? The answer appears to be more complex ( Figure 2). Overexpression of Bcl-2 in tumours can sensitise them to apoptosis when the function of Bcl-2 is inhibited, and in some cancers, high Bcl-2 expression is associated with better prognosis ^27^. In contrast, however, Mcl-1 is associated with poor prognosis in breast cancer ^28^. Cancer cells are generally more genetically unstable than normal cells and, as such, are much closer to the threshold for MOMP than healthy counterparts ^29^. Thus, the increased expression of anti-apoptotic proteins in these cells is required to suppress the pro-apoptotic effect of oncogenic transformation and genomic instability. Cancer cells that are dependent on overexpressed Bcl-2 are thus susceptible to its inhibition. This forms the basis for BH3-mimetics like venetoclax (discussed below).

Downregulation of pro-apoptotic Bcl-2 proteins is functionally equivalent to overexpressing the anti-apoptotic ones, and loss of expression of pro-apoptotic proteins is seen in a range of cancers. Loss of the Bim gene is present in renal cell carcinoma ^30^ and in coordination with downregulation of PUMA in samples of patients with Epstein–Barr virus ^31^. Deletion of PUMA is also found in about 40% of Burkitt lymphoma cases ^32^, and its downregulation is seen in a range of cancer types (for example, by microRNAs in glioblastoma) ^33^. The role of PUMA in the p53 response would fit with its downregulation promoting genetic instability and resistance to genotoxic therapies. Changes in the pro-apoptotic proteins that drive MOMP are also seen in cancer. Frameshift mutations in Bax are found in colon cancer ^34, 35^, and a loss of function due to silencing by miR-365 was seen in cutaneous squamous cell carcinoma ^36^. However, as with the anti-apoptotic proteins, mouse models where individual pro-apoptotic Bcl-2 family proteins have been deleted have not significantly predisposed those animals to tumours (summarised in 10). Gene deletion studies in mice show that Bax and Bak are largely redundant, and loss of one or the other has no overt oncogenic effect. Bad and Bid knockout mice are predisposed to lymphoma and myeloid tumours respectively, but again with a long latency. As with anti-apoptotic Bcl-2 proteins, loss of pro-apoptotic proteins can cooperate with other oncogenes to accelerate tumour development and progression.

## Exploiting the apoptotic landscape of cancer for therapy

The implication of altered Bcl-2 protein expression in tumours is that the sensitivity of those tumours to apoptosis in response to a variety of therapies will change. Recent developments allow changes in the Bcl-2 protein landscape of tumours to be exploited. The most promising approaches are based upon BH3-only protein specificity for binding the multidomain anti-apoptotic proteins and the changes in cancer cells that make them reliant on those anti-apoptotic proteins to stay alive ^37^. This understanding can be used both to test how a tumour’s Bcl-2 landscape determines how it will respond to chemotherapy and to therapeutically manipulate that landscape to sensitise or kill cancer cells.

Conventional chemotherapies kill cells via the mitochondrial pathway of apoptosis. Therefore, the myriad of variations in expression and function of pro- and anti-apoptotic Bcl-2 proteins combine in a cancer cell to determine how that cell responds to a drug designed to activate that process. Testing where that apoptotic threshold lies can predict therapeutic response before subjecting a patient to potentiality prolonged, ineffective and harmful treatments. BH3 profiling can measure mitochondrial priming in primary cancer cells isolated directly from patients ^38^. By exposing mitochondria from these cancer cells to peptides mimicking the functional domains of BH3-only proteins and measuring mitochondrial depolarisation as a surrogate readout for MOMP, it is possible to determine the apoptotic threshold of cells in a relatively rapid test. BH3-profiling therefore can identify cancer cells that are reliant on specific subsets of anti-apoptotic Bcl-2 proteins for survival, based upon the response to BH3-domain peptides with distinct binding specificities for those anti-apoptotic proteins ^39^. For example, primary samples from patients with acute myeloid leukaemia (AML) are significantly more primed than cells from healthy patients, as treatment with a Bim BH3-peptide causes significant apoptosis in the AML samples ^40^. AML cells that showed strong mitochondrial depolarisation in response to BH3-peptides came from patients who subsequently presented a better response to therapy than those whose cancer cells were relatively unresponsive. Similar results were found in other cancers, including lymphoma cell lines ^41^, primary acute lymphoblastic leukaemia (ALL) ^42^ and CLL ^43^.

The potential advantage of BH3-profiling is that the assay is based upon a functional test rather than an expressed biomarker which may not reflect a biological response. As there are a multitude of ways that cancer cells can alter Bcl-2 protein function, testing a limited set of expressed biomarkers may not predict patient response. Functionally testing this threshold does, provided that cancer cells can be isolated directly from the patient while not altering the Bcl-2 landscape. However, BH3-profiling requires cells to be dissociated, and how robustly it will predict responses in cancers where signals from the tumour microenvironment influence apoptotic priming remains to be seen.

Perhaps the most significant advance in applying knowledge of apoptotic regulation has been the development of BH3-mimetics. Venetoclax, the first drug in this class to be approved, is now in clinical use for treatment of CLL ^44^. Our understanding of how the Bcl-2 landscape sets apoptotic priming through the interaction of the different proteins within the family has highlighted points at which these interactions can be targeted. One specific vulnerability is the BH3-domain binding groove of anti-apoptotic Bcl-2 proteins. The initial report of ABT-737 in 2005 established the paradigm for these novel targeted drugs ^45^. A nuclear magnetic resonance (NMR)-based screen initially identified compounds that interacted Bcl-XL within the same hydrophobic groove within which BH3-only proteins bound, thus with the potential to compete for binding. These compounds were developed into ABT-737, a small molecule that shared the binding profile of the BH3-domain of Bad, with high affinity for Bcl-XL, Bcl-2 and Bcl-W but not with Mcl-1 or A1. As such, ABT-737 could compete with BH3-only proteins for binding to Bcl-XL, Bcl-2 and Bcl-W and thus increase apoptotic priming in cells. ABT-737 showed promise by inducing regression of solid tumours in xenografts of lymphoma and SCLC ^45^ as well as part of a combination therapy for head and neck squamous cell carcinoma ^46^.

The lack of oral bioavailability prevented ABT-737 from being clinically useful but led to the development of ABT-263 (marketed as navitoclax) ^47^. Navitoclax had essentially the same binding characteristics for anti-apoptotic Bcl-2 proteins as ABT-737 and was successful in inducing regression of SCLC and ALL xenografts in mice ^48^. However, acute thrombocytopenia was observed in multiple *in vivo* studies in mice, dogs and rats ^49^ as well as in clinical trials in patients with lymphoid tumours ^50, 51^. Although most adult tissues are remarkably tolerant of ABT-263, clinical testing identified a previously unknown role for Bcl-XL in platelet lifespan ^52^. Although initially this was a block on the use of navitoclax in patients, currently there are trials to test its safety by controlling thrombocytopenia through appropriate clinical management and dosing. Additionally, a previous clinical trial examining the effect of the aurora A kinase inhibitor MLN8237 showed a modest effect on solid tumours in combination with ABT-263 ^53^. As *BCL2L1* is one of the anti-apoptotic genes most often amplified in solid tumours, it will be important to therapeutically target this anti-apoptotic member despite the on-target side effects.

If specific tumours are dependent upon Bcl-2, then BH3-mimetics with a single-molecule specificity would be desirable to minimise adverse effects. Modifying ABT-263 to increase its specificity for Bcl-2 resulted in ABT-199 ^54^. ABT-199 was trialled in CLL, where it showed a remarkable effectiveness as a single agent ^55^. Indeed, ABT-199 was so effective in some cases that it caused potentially fatal tumour lysis syndrome, subsequently controlled by changes in dosing. ABT-199 (marketed as venetoclax) has been approved as a single agent for treatment of CLL patients with a 17p deletion who have had at least one prior therapy ^44^.

Although CLL shows significant dependency on Bcl-2, other cancers are not as sensitive to inhibiting it alone. In November 2018, venetoclax was granted accelerated approval by the US Food and Drug Administration for the treatment of AML in combination with zacitidine, decitabine or low-dose cytarabine in adults who are 75 or older or who have comorbidities that prevent the use of other treatments. There are numerous studies under way examining the use of ABT-199 either alone or in combination in the treatment of multiple other cancer types. The majority of these are still in early stages, but ** phase III trials are examining long-term safety data for the use of venetoclax in treating CLL and AML as well as multiple myeloma and ALL. There is also a phase III trial investigating the effects of venetoclax in combination with ibrutinib to treat mantle cell lymphoma. A more recent study examining the addition of venetoclax for treatment of pancreatic cancer has shown inhibition of growth in xenograft mouse models to be more effective when both drugs are used in combination ^56^. Venetoclax has also shown promise in combination with histone deacetylase (HDAC) inhibition in treating multiple myeloma ^57^. Thus, BH3-mimetics will undoubtedly be used not just to target tumours where Bcl-2 dependency is keeping them alive but to shift the apoptotic landscape to make them more primed for MOMP and thus susceptible to other treatments. This concept has been tested with what is termed dynamic BH3-profiling, where response of isolated cancer cells to BH3-peptides was tested in conjunction with other targeted cancer therapies ^38^.

However, it is not only Bcl-2 which is aberrantly regulated in cancer. Although CLL is largely Bcl-2–dependent, other cancers use a combination of anti-apoptotic proteins to survive, requiring either multiple BH3-mimetics or ones with broader binding characteristics. As detailed by the National Cancer Institute ( https://www.cancer.gov), navitoclax, alone or in combination with other drugs, is still under investigation for use in a number of malignancies, including relapsed small cell lung cancer and metastatic melanoma. Trials of navitoclax with rutiximab for lymphoid malignancies are ongoing ^58^. Bcl-XL–specific mimetics have been developed ^59^, and there is still significant need in targeting this appropriately in Bcl-XL–dependent tumours. Mcl-1 is also a target of interest due to its expression in numerous cancers, such as breast tumours with poor outcomes ^28^. Although Mcl-1 initially proved a difficult target to develop specific mimetics for a number of years, potent inhibitors have now been developed ^60– 63^, of which S63845 from Servier shows the greatest specificity over Bcl-2 and Bcl-XL ^64^. These compounds are undergoing trials. Furthermore, small molecules have been developed that, rather than inhibiting anti-apoptotic Bcl-2 proteins, directly activate pro-apoptotic ones such as Bax ^65, 66^. Intriguingly, the Bax-activating compound BTSA1 was found to kill AML cells while being tolerated by healthy cells, both *in vitro* and *in vivo*. Overall, the area of BH3-mimetics and related drugs is expanding and offers the exciting potential of targeting apoptosis directly in cancer.

## Acquired resistance to BH3-mimetics: the apoptotic landscape as a moving target

Venetoclax it is not a panacea for cancer therapy. A common problem with many targeted drugs against tumour-specific mutations is acquired resistance, seen with imatinib in patients with chronic myeloid leukaemia (CML) ^67^, vemurafenib in melanoma ^68^ and cetuximab in colorectal cancer ^69^. Acquired resistance is often due to *de novo* mutations in the drug targets that impede the drugs’ mechanism of action. For example, acquired *BCR-ABL* mutations in patients with CML block imatanib binding to the kinase domain. Similarly, a single amino-acid substitution in the extracellular region of the epidermal growth factor receptor prevents cetuximab from binding in colorectal cancer patients presenting with acquired resistance. Thus, given the highly targeted nature of venetoclax, the emergence of acquired resistance would be expected. Indeed, mechanisms analogous to those seen with other targeted drugs have been identified both experimentally and in clinical trials.

Studies specifically tried to anticipate the emergence of resistance to BH3-mimetics using a murine mantel cell lymphoma (MCL) model. MCL lines were exposed to increasing doses of ABT-737 and ABT-199 over several months until resistant cells emerged ^70^. Drug resistance was seen both *in vitro* and *in vivo* and was maintained even when cells were subsequently cultured in the absence of drug and then re-exposed. The resistance was specific to the BH3-mimetics, as the cells readily underwent apoptosis when treated with other anti-cancer drugs. Intriguingly, one human MCL line showed acquired resistance to a wider range of anti-cancer drugs, including taxol and doxorubicin. The study authors found that two distinct mutations had been acquired in the mouse and human cells. In the mouse lines, two novel missense mutations in Bcl-2 itself had emerged, resulting in amino-acid substitutions within the BH3-binding pocket (F101C and F101L). These two substitutions resulted in significantly reduced binding of both ABT-737 and ABT-199 to the variant Bcl-2. In contrast, the resistant human MCL line had acquired a missense mutation in the pro-apoptotic effector Bax, specifically an amino-acid substitution within its mitochondrial targeting sequence leading to a more general reduction in sensitivity to pro-apoptotic drugs.

Although trials with CLL patients who had undergone previous drug treatments showed a good response to venetoclax, disease progression occurred in a number of patients after 2 to 3 years on the BH3-mimetic ^44^. Two independent studies found that this acquired resistance in a number of patients on long-term venetoclax treatment resulted from mutations in the *BCL-2* gene, in the same region as those identified in experimentally induced resistance in the murine study ^71, 72^. In one study ^71^, seven patients with recurrent venetoclax-resistant CLL were found with a glycine-to-valine substitution at position 101 within the BH3-binding pocket. The patients’ tumours did not have this variant prior to venetoclax treatment. The mutations were first detected at a low frequency after many months of drug exposure and preceded disease recurrence by several months. The second study ^72^ found the same G101V substitution in four patients with recurrent CLL, one of which had tumour cells with a second, independent Bcl-2 variant containing a substitution of aspartate 103 to tyrosine, again within the BH3-binding pocket.

In both the experimental and clinical occurrences of venetoclax resistance, the Bcl-2 variants identified significantly reduced binding to ABT-737 and ABT-199 but had minimal effect on binding to pro-apoptotic Bcl-2 proteins such as Bax and Bim ^71, 73^. As such, the specific variants selected for in patients resulted in reduced drug sensitivity whilst maintaining Bcl-2 anti-apoptotic function. Recent structural analysis of the G101V, F104C and F104L variants has indicated that they all reduce ABT-199 binding but through distinct mechanisms ^73^. This indicates that despite their well-defined mechanism of action, BH3-mimetics do not fully recapitulate the binding of full-length BH3-only proteins to their anti-apoptotic partners. Interestingly, a number of recent *in vitro* studies have led to similar conclusions. One recent study found that Bim bound to Bcl-XL via two separate interactions, one of which is the classic BH3-groove ^74^. This dual lock-and-key binding means that Bim is more resistant to competition with ABT-263 than might be expected. In another study, the membrane interaction of Bcl-XL increased its affinity for the BH3-only proteins PUMA and Bim, again resulting in resistance to competition by ABT-263 that was not seen with a soluble version of Bcl-XL ^75^. Since the isolation of ABT-737 and the clinical adoption of venetoclax, other BH3-mimetics which show binding specificities for distinct Bcl-2 family members, such as S55746, have been generated ^76^. The potential for distinct binding properties suggests that it is possible overcome the acquired venetoclax resistance that is emerging, and the structural information on venetoclax-resistant Bcl-2 variants will provide a rational basis for drug optimisation ^73^. However, there are clearly still some aspects of the underlying biology that need to be understood.

## Dynamic heterogeneity of apoptosis in healthy tissues and cancer

Apoptotic priming in normal cells and tissues varies dynamically at multiple levels. This variation can be seen between different adult tissues, within the same tissue at distinct developmental stages, between individual cells within a tissue, and in the same cell at different times as that cell is exposed to damage or changes in signalling. The apoptotic landscape is constantly shifting, which presents difficulties when targeting cancer cells whilst minimising the damage to healthy tissue.

During embryonic development, where significant levels of apoptosis occur, changes in Bcl-2 protein function are linked to distinct developmental events. In the developing nervous system of mice, neuronal Bcl-2 levels significantly decrease by 5 months of age ^77^. Bcl-XL and Mcl-1 play key roles in the survival of immature B cells during development, whereas Bcl-2 is required for survival of mature B cells ^78^. The shifting balance of priming during development is also influenced by changes in pro-apoptotic Bcl-2 proteins, although as discussed above in relation to cancer predisposition, individual BH3-only proteins seem not to have significant roles in the development of most tissues. However, a recent study found that subtle changes in the balance of pro- and anti-apoptotic Bcl-2 proteins have profound effects during craniofacial development ^79^.

Changes in the apoptotic threshold are also apparent post-natally and during development into adults. A recent study used BH3-profiling to compare apoptotic priming in paediatric and adult tissues ^80^. The results showed that many adult tissues are profoundly insensitive to apoptosis, and mitochondria from these tissues showed low responses to a broad panel of BH3-only protein peptides. In contrast, many of the same embryonic and post-natal tissues were sensitive to mitochondrial apoptosis. During post-natal development, the overall landscape of many Bcl-2 proteins changes, altering the sensitivity of these tissues, with a general reduction in expression of Bax and Bak with age ^80^. This explains not only why many adult tissues show a broad tolerance to the new BH3-mimetics and many genotoxic drugs but also why children are more at risk of toxic side effects to chemotherapy. Changes in age-related apoptotic priming were linked to c-Myc, which is expressed in developing tissues and has been linked to changes in apoptotic sensitivity. Forced expression of c-Myc in adult mouse brain increased the apoptotic response to genotoxic damage. This response occurred at a single-cell level, and those cells expressing c-Myc showed increased sensitivity to apoptosis. Thus, apoptotic priming can be set at the level of the individual cell, a further level of heterogeneity that impacts on cancer therapy.

Many of the signalling pathways that control cell proliferation and Bcl-2 protein function also show single-cell heterogeneity in their activity ^81, 82^. Therefore, it should not be surprising that apoptosis shows significant cell-to-cell variation. Single cells can also shift their apoptotic threshold rapidly in response to changes in internal and external signals ^83^. One way this can occur is through Bcl-2 proteins continuously shuttling between cytosol and mitochondria, buffering cells against tolerable levels of stress signalling ^83^. Where the apoptotic threshold of individual cells is dynamic, the cell population reaches an equilibrium between sensitivity and resistance to pro-apoptotic stimuli ^84^. Subsequently, the outcome following exposure to an apoptotic signal varies significantly within and between cell populations ^85^. Thus, targeting cancer cells for apoptosis via disrupting Bcl-2 protein interactions is complicated as our current knowledge of these single-cell dynamics is limited. Although predictive tools such as BH3 profiling provide an indication as to treatment outcome at a tissue level, such tests are only a snapshot in time of the population response and may not predict levels of resistance to specific drug combinations.

However, this ability of cells to rapidly shift their apoptotic sensitivity has the potential to be exploited through approaches such as dynamic BH3 profiling ^38^. This allows the effect of targeted drugs, such as kinase inhibitors, on apoptotic threshold of cancer cells to be determined. Many of these drugs target the dynamic signalling pathways that post-translationally alter Bcl-2 protein function through phosphorylation, degradation, or by altering their sub-cellular localisation. With this approach, it may be possible to screen a primary tumour sample for those targeted drugs that increase apoptotic priming. For example, BH3-profiling was able to discriminate CML patients who showed a response to imatinib or not ^38^.

Overall, our understanding of how apoptosis is controlled in mammalian cells has reached a level of maturity whereby it can be rationally applied in a clinical setting. However, there are still levels of complexity in understanding how it is dynamically controlled within single cells and populations of those cells. This needs to be explored in more depth in order to maximise how we can manipulate cell death therapeutically.
